# Welcome to the first issue of *BJR|case reports*!

**DOI:** 10.1259/bjrcr.20150193

**Published:** 2015-05-12

**Authors:** G Guglielmi

**Affiliations:** ^1^Department of Radiology, University of Foggia, Foggia, Italy; ^2^Department of Radiology, Scientific Institute “Casa Sollievo della Sofferenza” Hospital, Foggia, Italy

As Editor-in-Chief I am delighted to welcome you to the first issue of *BJR|case reports*, the British Institute of Radiology’s (BIR) new open access, online-only case reports journal with a broad appeal to radiologists, radiation oncologists and researchers in the radiation sciences.

Case reports are an important vehicle for presenting interesting and unusual clinical information and treatment decisions. *BJR|case reports* also accepts technical notes to capture the first stages of technical advances being made in the radiation sciences. For readers, case reports and technical notes are especially important as teaching tools, helping to put the presentation of a patient into a broader context; and for authors they are many researchers’ first step on the ladder of their publishing career.

With many research journals deciding no longer to publish case reports, *BJR|case reports *has been launched to create a dedicated home for this kind of article. The BIR, with a history going back more than a century of working with the wider radiological community and publishing the latest research, is the natural home for a journal of this kind.

All *BJR|case reports* articles are published under an open access licence and will always be free for anyone to access and re-use the content, with correct attribution. This means that articles will reach the widest possible audience and with continuous publication used, be available online as soon as they are ready.

After an overwhelmingly positive response following the launch of the journal at the end of 2014, *BJR|case reports* published its first articles in March 2015 and now follows with the first issue of this exciting new radiological resource.

Issue 1 of our quarterly journal adds to the spread of content published so far, containing case reports covering a range of clinical systems and imaging modalities including lung, breast, gastrointestinal and vascular imaging, as well as nuclear medicine and interventional procedures.

Supporting me in my role as Editor-in-Chief is an international Editorial Board to oversee the rigorous peer review of the submissions, and we have been pleased to see the community embrace the journal and submit their work. *BJR|case reports* has so far received submissions from 28 countries and our first issue demonstrates the international appeal of the journal, with authors from the United States, India, Greece, Australia and the United Kingdom.

I believe *BJR|case reports* represents a fantastic opportunity for clinicians and researchers starting to venture in to scientific publishing, by writing up and submitting their first case report and being able to familiarise themselves with the freely accessible published content.

I look forward to the exciting times ahead growing and developing the journal into an open resource and destination for novel, original and educational case reports for the radiology and radiation sciences community.

We look forward to receiving your case report soon!

